# Zika virus diversity in mice is maintained during early vertical transmission from placenta to fetus, but reduced in fetal bodies and brains at late stages of infection

**DOI:** 10.1371/journal.pntd.0011657

**Published:** 2023-10-05

**Authors:** Alyssa B. Evans, Clayton W. Winkler, Sarah L. Anzick, Stacy M. Ricklefs, Dan E. Sturdevant, Karin E. Peterson

**Affiliations:** 1 Laboratory of Neurological Infections and Immunity, Neuroimmunology Section; Rocky Mountain Laboratories; National Institute of Allergy and Infectious Diseases (NIAID); National Institutes of Health (NIH); Hamilton, Montana, United States of America; 2 Genomics Research Section, Research Technologies Branch; Rocky Mountain Laboratories; National Institute of Allergy and Infectious Diseases (NIAID); National Institutes of Health (NIH); Hamilton, Montana, United States of America; Texas A&M University, UNITED STATES

## Abstract

Since emerging in French Polynesia and Brazil in the 2010s, Zika virus (ZIKV) has been associated with fetal congenital disease. Previous studies have compared ancestral and epidemic ZIKV strains to identify strain differences that may contribute to vertical transmission and fetal disease. However, within-host diversity in ZIKV populations during vertical transmission has not been well studied. Here, we used the established anti-interferon treated *Rag1*^-/-^ mouse model of ZIKV vertical transmission to compare genomic variation within ZIKV populations in matched placentas, fetal bodies, and fetal brains via RNASeq. At early stages of vertical transmission, the ZIKV populations in the matched placentas and fetal bodies were similar. Most ZIKV single nucleotide variants were present in both tissues, indicating little to no restriction in transmission of ZIKV variants from placenta to fetus. In contrast, at later stages of fetal infection there was a sharp reduction in ZIKV diversity in fetal bodies and fetal brains. All fetal brain ZIKV populations were comprised of one of two haplotypes, containing either a single variant or three variants together, as largely homogenous populations. In most cases, the dominant haplotype present in the fetal brain was also the dominant haplotype present in the matched fetal body. However, in two of ten fetal brains the dominant ZIKV haplotype was undetectable or present at low frequencies in the matched placenta and fetal body ZIKV populations, suggesting evidence of a strict selective bottleneck and possible selection for certain variants during neuroinvasion of ZIKV into fetal brains.

## Author summary

Zika virus (ZIKV) is known to cause congenital disease in humans through vertical transmission of the virus from mothers to fetuses during pregnancy. How the virus populations change during this process is currently not well understood. To evaluate differences in ZIKV populations during vertical transmission, we infected pregnant mice with ZIKV and sequenced the viral populations from matched placentas and fetal bodies at early stages of vertical transmission, and matched placentas, fetal bodies, and fetal brains at late stages of vertical transmission when the virus enters the fetal brain. We determined that there is little restriction of ZIKV diversity during transmission from the placenta to the fetus at early stages. However, there was a sharp decrease in ZIKV diversity in both fetal bodies and fetal brains at late stages. Two predominant viral variants were found across all fetal brains and most fetal bodies at late stages, suggesting there may be possible selection for certain variants during neuroinvasion of ZIKV into fetal brains.

## Introduction

Zika virus (ZIKV) emerged in Brazil in 2015 as a mediator of severe congenital complications in human fetuses, including microcephaly [1,2]. Since then, considerable research has focused on this single-stranded, 10.8kb, positive sense RNA member of the *Flaviviridae* family. ZIKV was first implicated as an important human pathogen in 2007, when an epidemic broke out on Yap Island and Guam [3]. In 2013, French Polynesia experienced a ZIKV epidemic and for the first time ZIKV was associated with neurological complications [4,5]. During the Brazilian and Americas outbreak in 2015–2016, a causative link between ZIKV and microcephaly was established. Other brain and fetal abnormalities have since been described and are collectively known as congenital ZIKV syndrome (CZS); [2,6].

A great deal of research has been conducted in the past several years to evaluate ZIKV lineage differences to identify if there were lineage-specific differences or mutations that resulted in the neurological disorders associated with ZIKV outbreaks of the Asian lineages in French Polynesia and Brazil. It was initially suggested that Asian lineages had mutations conferring increased neurovirulence and fetal demise compared to African lineages [7]. However, a growing body of evidence has shown that both Asian and African lineages are capable of causing severe disease with adverse fetal outcomes [8]. There is some evidence that the African lineage may be more virulent [9,10], but strain-specific differences in virulence also exist [11]. Therefore, vertical transmission and neurovirulence may be a largely shared trait across multiple ZIKV lineages and strains. It is possible then that pathogenic outcomes from ZIKV infection are, at least in part, mediated by within-host ZIKV variation.

Less is known about within-host ZIKV diversity, and how that diversity may impact vertical transmission and subsequent fetal disease. In one study of ZIKV diversity in non-pregnant and pregnant macaques, a barcoded ZIKV was used to analyze ZIKV population diversity over time [12]. The authors found that in both the pregnant and non-pregnant macaques, ZIKV diversity in the serum remained stable during the course of acute infection. However, in a single pregnant macaque, ZIKV diversity in the serum decreased following the acute phase. Unfortunately, ZIKV was not recovered in any fetal tissues, and therefore ZIKV diversity in the fetus could not be evaluated [12]. In a separate study evaluating ZIKV variation during pregnant macaque infection, a single nucleotide variant was identified from whole genome sequencing in maternal tissues of a single pregnant macaque, and further sequencing analysis of pregnant macaques and mice was only performed targeting that variant [13]. Another study showed that ZIKV diversity in infected mice varied by organs and inoculation routes; however only non-pregnant animals were used, so again fetal tissues were not evaluated [14]. A recent study evaluated ZIKV diversity in a pregnant pig via in utero inoculation of individual conceptuses and identified some tissue-specific variants as well as a low level of convergent evolution [15]. Unfortunately, in that study, none of the fetal brains were positive for ZIKV at the study endpoint so variants involved in neuroinvasion could not be evaluated [15]. To our knowledge, no study thus far has evaluated whole genome ZIKV diversity within animals during early and late stages of vertical transmission and fetal neuroinvasion.

To evaluate ZIKV diversity during vertical transmission and entry into the fetal central nervous system (CNS), we used the previously established anti-interferon treated *Rag1-/-* (AIR) mouse model of ZIKV vertical transmission, which was associated with higher fetal viability than the *Ifnar-/-* x WT model [16,17]. We inoculated pregnant AIR mice with ZIKV and evaluated the ZIKV populations in matched placentas, fetal bodies, and fetal brains by RNASeq to determine the pattern of ZIKV transmission from the placenta to the fetus and the fetal brain. We compared placenta and fetal body ZIKV populations at early stages of vertical transmission and fetal infection, as well as ZIKV diversity in placentas, fetal bodies, and fetal brains at later stages of fetal infection to determine if there were differences in ZIKV diversity within tissues and if certain ZIKV variants were associated with vertical transmission and entry into the fetal brain. Our findings indicate that during early stages of ZIKV vertical transmission there is little restriction in ZIKV diversity between the placenta and fetus. This is followed by a sharp reduction in ZIKV diversity in the fetal periphery and brain at later stages of fetal infection, with evidence of possible selection for certain ZIKV variants in the fetus.

## Results

### ZIKV transmits from the placenta to the fetal periphery before subsequent transmission into the fetal brain

One of the critical components for ZIKV-induced microcephaly is the spread of virus from placenta to the fetal periphery and fetal brain. In our previous studies of ZIKV vertical transmission using the AIR mouse model, ZIKV was consistently detected in placentas and fetal brains, and in some fetal lymph nodes [18]. Further studies showed that infection in the fetal periphery likely occurred prior to infection in the brain in the 11–12 days post inoculation (dpi) time frame [19]. To examine more specifically the timing of ZIKV transmission between the placenta, fetal periphery, and fetal brain, 7 pregnant AIR dams were inoculated with ZIKV and necropsied at 7, 11, 13, or 14 dpi. Due to the three-day mating window, these time points corresponded to embryonic days (E) ~11–14 (7 dpi), 17 (11 dpi), 17–18 (13 dpi), and 19 (14 dpi). ZIKV RNA was analyzed in placentas, whole fetuses (7 dpi), or fetal bodies and fetal heads (11–14 dpi).

**Fig 1:**
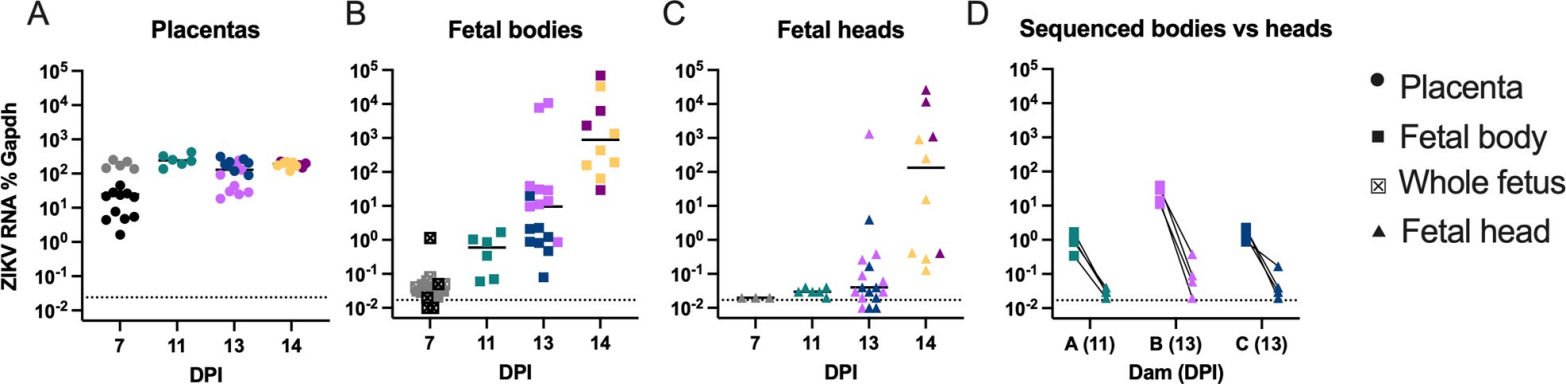
Time course of ZIKV RNA levels in placentas, fetal bodies, and fetal heads during vertical transmission. Pregnant AIR mice were inoculated at 7 days post-mating. A-D) At 7, 11, 13, and 14 dpi, mice were euthanized and tissues analyzed by RT-qPCR for ZIKV RNA in (A) placentas, (B) whole fetus or fetal bodies, and (C) fetal heads. D) ZIKV RNA levels in fetal bodies selected for sequencing and the matched head (not sequenced) is plotted with lines connecting tissue from the same fetus. Dashed lines denote mock-inoculated levels in the tissues. Each dam is represented by a different color, and each tissue by an individual symbol.

All placentas, regardless of dpi, had high levels of ZIKV RNA (Fig 1A). For both fetal bodies and fetal heads, the amount of ZIKV RNA tended to increase over time, although there was variation between dams and fetuses (Fig 1B and 1C). All fetal bodies had detectable levels of ZIKV RNA by 11 dpi (Fig 1B). No fetal heads had ZIKV RNA levels different from mock at 11 dpi, and only about half of the fetal heads at 13 dpi had moderate to high levels of ZIKV RNA (Fig 1C). All fetal heads had detectable ZIKV RNA by 14 dpi. We could not perform plaque assays on these samples as RNA was isolated in TRIZOL in anticipation of use in RNASeq, therefore we do not know the infectious titers for each sample. However, from our previous studies using this model, ZIKV RNA levels in placentas, fetal bodies, and fetal heads strongly correlated with infectious virus titer via plaque assay [19]. Overall, the increase in ZIKV RNA in fetal heads appeared delayed compared to the fetal bodies, consistent with previous results [19] Fig 1D).

The moderate variation of ZIKV RNA in the fetal bodies and brains between fetuses from the same dam as well as fetuses between different dams is likely due to the variability in pregnancy and embryonic day of development when the dams were inoculated with ZIKV, as mating pairs were set up for a three-day window to increase the chances of pregnancy. The first anti-interferon receptor 1 (IFNAR1) antibody treatment was given at six days post initial mating set up (E ~3–6) and female mice were inoculated at seven days post initial mating set up (E ~4–7). While the timing of anti-IFNAR1 antibody treatment and ZIKV inoculation was consistent between mice, the embryonic stage at the time of these treatments and inoculation could affect the kinetics of infection and transmission to the fetus. While ZIKV RNA in the placentas was consistently high regardless of dpi (Fig 1A), the susceptibility of fetuses to infection or the ability of the virus to transmit from the placenta to the fetus may differ between developmental days. However, ZIKV RNA levels were consistently higher and detected earlier in fetal bodies compared to fetal brains, confirming that ZIKV first enters and replicates in the fetal periphery prior to entering the brain during ZIKV vertical transmission. In our model, we found that transmission of ZIKV from the placenta to the fetal periphery began in some fetuses by ~7 dpi, and neuroinvasion from the fetal periphery to the fetal brain happened at ~13 dpi.

### There is little restriction in ZIKV diversity during transmission from placenta to fetus

Mice, like humans, have discoid hemochorial placentas where each fetus has its own discreet placenta [20]. Therefore, we were able to compare viral variants in matched placentas and fetuses. Previous studies in the AIR mouse model demonstrated that the maternal side of the placenta is the primary site of ZIKV replication in the placenta [16], and that vertical transmission of ZIKV occurs via an active mechanism, rather than breakdown and leakage of the blood-placenta barrier [16].

We therefore wanted to analyze if ZIKV transmission across the placenta into the fetus was limited to certain ZIKV variants. To do this, we sequenced ZIKV viral populations via RNASeq in matched placentas and fetal bodies and compared the viral populations between the matched tissues. We wanted to sequence viral populations at the early stages of vertical transmission soon after ZIKV entered the fetus to identify the ZIKV variants that transmitted from the placenta to the fetus. This limited the time the virus had to replicate within the fetus and either acquire additional mutations or lose transmitted variants. We therefore selected four placentas and fetal bodies each from one of three different dams (A, B and C) with moderate levels of ZIKV RNA, but with low or undetectable levels of ZIKV RNA in the corresponding fetal heads (Fig 1D). This suggests that ZIKV in these samples would have gone through limited rounds of replication in the fetus post-transmission. We also sequenced our ZIKV inoculum stock.

Sequence reads were aligned to the Paraiba reference strain (GenBank: KX280026.1) using Bowtie2 and single nucleotide variants were detected using Genome Analysis Tool Kit (GATK) [21]. Variants were manually reviewed and confirmed in Integrative Genomics Viewer (IGV) [22]. We used a variant frequency threshold of 10%, so that variants identified by GATK were reported if a sample had a nucleotide that differed from the reference strain in ≥10% of the reads at that position. Sites that differed from the reference in 10–90% of the reads for a given sample are designated “variable sites” and sites with a variant in >90% of reads are designated “dominant variants”.

The inoculum had >3000x coverage, placentas had >1000x coverage, and the fetal bodies had 5-14x coverage. The inoculum contained five variable sites, however all five positions reverted back to 100% reference nucleotide in all of the placenta populations (S1 Table). The inoculum virus was passed at least twice in cell culture, and these results indicate that the inoculum acquired cell-culture adapted mutations, but these reverted to wild-type under selection pressures *in vivo* when inoculated into mice.

Individually, the fetal bodies had low ZIKV sequence coverage due to the vast majority of sequencing reads mapping to the host genome. Low sequence coverage for viral variant calling can inflate minor variant frequencies or miss real variants and thus distort the true variation within a population. To more accurately compare variants and ZIKV population structures between the placentas and fetal bodies, reads from the four fetal bodies and placentas from each dam were combined, bringing the fetal body mean ZIKV coverage to 33-46x, and placenta mean coverage to >3000x (S1 Table). We then identified sites with ≥10% of reads with a different nucleotide from the reference in the placentas or fetal body populations. There were 27 nucleotide positions that met the ≥10% threshold in placentas and/or fetuses from at least one dam (S1 Table). Additionally, from our second set of sequencing of later stages of vertical transmission (described below), we identified four additional variable sites. We then designed specific primers to target all 31 variable sites in all individual samples in a targeted sequencing approach to increase sequencing coverage. We were able to successfully amplify and sequence 29 of the variable sites from 13 primer sets with mostly non-overlapping amplicons of lengths of 400–411 base pairs (bp), for a total genome coverage of ~5260bp. The other two variable sites, at positions 2471 and 9722 and both only found in dam C (S1 Table) could not be amplified.

**Fig 2:**
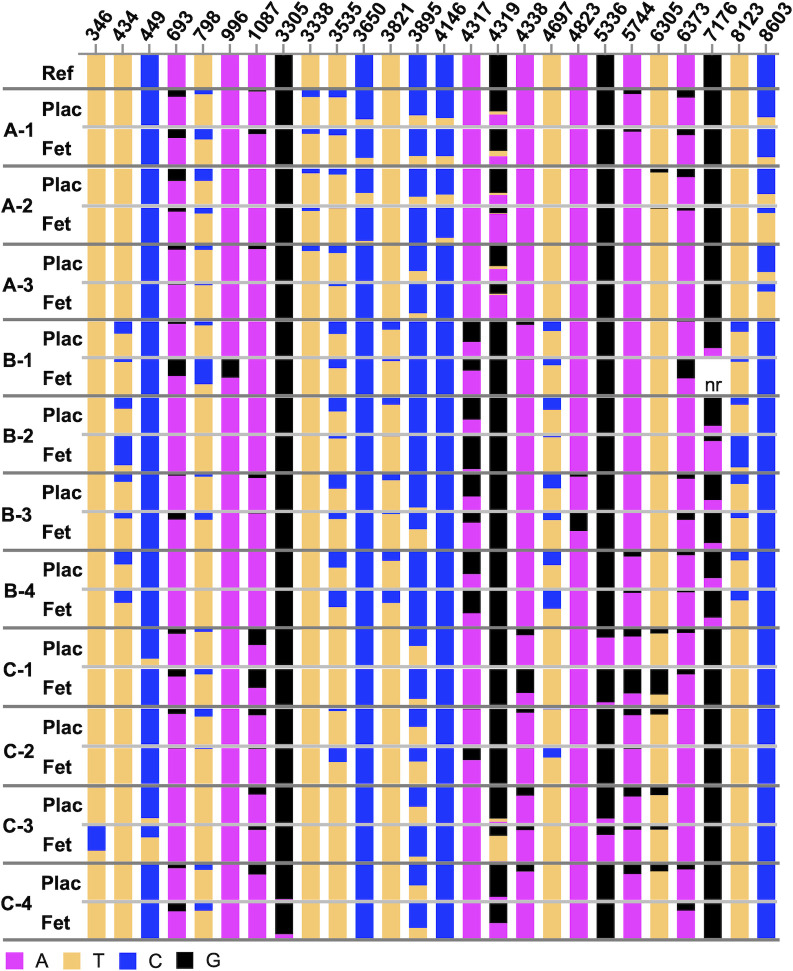
Graphical representation of variable sites from targeted sequencing of early stages of vertical transmission. Sequencing data summarized in Tables 1–3 is shown. All variable sites across all three dams are plotted, with each nucleotide represented by a different color. Ref indicates the reference nucleotide, and proportions of reference vs. alternate allele are represented in each bar graph. Plac = placenta, Fet = fetal body, nr = no reads. Matched tissues are separated by light gray lines, different samples are separated by dark gray lines.

The targeted site primer sets were used to sequence all 12 matched placentas and fetuses from the three dams from this set. This approach resulted in average sample coverage for targeted sites of 176-210x for placentas and 147-201x for fetal bodies. One fetal body, A-4, had an average coverage of 12x with poor quality scores from the targeted sequencing, and therefore it and its matched placenta were excluded from analysis. The targeted results are summarized for each dam in Tables 1–3 and graphically displayed in Fig 2. Results largely matched the pooled results from the whole genome sequencing (S1 Table). Variant frequencies were reported for the targeted sites, as well as any non-targeted site covered by the primers, that reached ≥10% threshold in a sample, and the corresponding variant frequency in the matched tissue regardless of frequency (Tables 1–3).

**Table 1:** Variant frequencies from targeted sequencing for Dam A.

							Dam A Targeted					
							A-1		A-2		A-3	
	Nucleotide			Amino acid			Plac	Fet	Plac	Fet	Plac	Fet
Gene	Pos	Ref	Alt	Pos	Ref	Alt	186x	189x	185x	147x	187x	194x
prM	693	A	G	196	T	A	19.7	25.3	35	10.5	11.8	*6*.*3*
M	798	T	C	231	S	P	12.6	29.6	34.5	16.1	12.4	*7*.*7*
Env	1087	A	G	327	D	G	4.8	14.5			10.4	2.1
NS1	3338	T	C	1077	T		20.3	13.8	13.2	*8*.*7*	15.5	<1
NS1	3535	T	C	1143	V	A	21.3	18	16.8	<1	21.5	10.1
NS2A	3650	C	T	1181	S		16.7	18.5	31.6	*6*.*6*		
NS2A	3895	C	T	1263	A	V	27.9	23.8	20.6	<1	27.8	13.4
NS2A	4146	C	T	1347	L		20.3	23.1	26.9	15.2		
NS2B	4319	G	A	1404	M	I	30.5	23	25.8	83.8	33.8	65.1
			T				8.4	15.3	5.7	1.1	7.9	3.2
NS3	5744	A	G	1879	T		12.1	*7*.*5*				
NS3	6305	T	G	2066	D	E			10.5	1.9		
NS3	6373	A	G	2089	K	R	22.2	17.3	24	*7*.*7*		
NS5	8603	C	T	2832	A		22.5	19.9	28	85.5	25.2	74.7
quality score	≥90					# variable sites (10-90%):	11	11	11	5	8	4
	*≤89*					# dominant variant (≥90%):	0	0	0	0	0	0

x = average coverage per position of targeted sites

**Table 2:** Variant frequencies from targeted sequencing for Dam B.

							Dam B Targeted							
							B-1		B-2		B-3		B-4	
	Nucleotide			Amino acid			Plac	Fet	Plac	Fet	Plac	Fet	Plac	Fet
Gene	Pos	Ref	Alt	Pos	Ref	Alt	189x	187x	181x	177x	209x	201x	176x	186x
Capsid	434	T	C	109	S		35.2	*7*.*9*	30.8	83.2	21.1	15.4	35.5	34.4
prM	693	A	G	196	T	A	*7*.*3*	47.9			3.8	18.4		
M	798	T	C	231	S	P	11.4	71.6			*6*.*7*	19.1		
Env	996	A	G	297	S	G	0	52.9						
Env	1087	A	G	327	D	G					10.3	1.4		
NS1	3535	T	C	1143	V	A	36.1	25.7	39.4	8.4	40.4	17.1	44.3	46.7
NS2A	3821	T	C	1238	S		23.6	*5*.*5*	19.7	1.8	16.8	2.9	25.2	34.8
NS2A	3895	C	T	1263	A	V					*6*.*9*	54.5		
NS2B	4317	A	G	1404	M	V	59.4	33	62.6	**94.1**	62.1	27.3	62.7	63.8
NS2B	4338	A	G	1411	I	V	10.1	1.6						
NS3	4697	T	C	1530	R		27.3	17.5	34.1	4.9	39.6	19.9	37.5	50.8
NS3	4823	A	G	1572							6.7	50.7		
NS3	5744	A	G	1879	T								13.2	6.5
NS3	6373	A	G	2089	K	R	10.4	54.6			12.5	19.3	10	*5*.*2*
NS4B	7176	G	A	2357	A	T	22.7	nr	19.7	84.4	28	15.2	26.1	23.7
NS5	8123	T	C	2672	S		29.9	*7*.*3*	19.8	89	27.2	14.3	24.2	27.9
quality score	≥90					variable sites (10–90%)	10	7	7	3	9	11	9	7
	*≤89*					**dominant variant (≥90%)**	0	0	0	1	0	0	0	0

x = average coverage per position of targeted sites

nr = no reads

**Table 3:** Variant frequencies from targeted sequencing for Dam C.

							Dam C Targeted							
							C-1		C-2		C-5		C-6	
	Nucleotide			Amino acid			Plac	Fet	Plac	Fet	Plac	Fet	Plac	Fet
Gene	Pos	Ref	Alt	Pos	Ref	Alt	202x	179x	188x	189x	189x	188x	210x	183x
Capsid	346	T	C	80	I	T					<1	69.5		
Capsid	449	C	T	114	G		17.6	0			13.3	67.2		
prM	693	A	G	196	T	A	13.3	20.7	15	1.1			11.3	24
M	798	T	C	231	S	P	*7*.*1*	14.9	22.2	2.7			15.7	22.2
Env	1087	A	G	327	D	G	44.4	52.9	18.9	2.4	20.3	11.9	28.6	0
NS1	3305	G	A	1066	E								*1*.*4*	*10*.*8*
NS1	3535	T	C	1143	V	A			6.8	38.9				
NS2A	3895	C	T	1263	A	V	53.1	16.6	48.9	62.6	45.7	13.6	40.6	29.1
NS2B	4317	A	G	1404	M	V			1.7	34				
NS2B	4319	G	A	1404	M	I					3.7	<1	*8*.*2*	42.4
			T								8.5	70.7	*0*	0
NS2B	4338	A	G	1411	I	V	17.4	66.7	11.6	1	23.3	12.2	20.3	0
NS3	4697	T	C	1530	R				1.7	26.5				
NS3	5336	G	A	1743	V		*76*.*5*	*6*.*9*			12.3	74.1		
NS3	5744	A	G	1879	T		*20*.*4*	68.6	18.6	2.5	26.2	11.4	27.3	0
NS3	6305	T	G	2066	D	E	12.3	71.7	16.3	0	22	10.9	19.6	<1
NS3	6373	A	G	2089	K	R	10.6	15.1					14.2	22
quality score	≥90					# variable sites (10-90%):	9	8	7	4	8	9	8	6
	*≤89*					# dominant variant (≥90%):	0	0	0	0	0	0	0	0

x = average coverage per position of targeted sites

Overall, results from both the whole genome and targeted approaches were consistent and showed that the ZIKV populations in the fetal bodies were similar to the populations in the placentas. More than 90% of identified variants were detected in both matched tissues at this early stage of fetal infection. Across all 11 matched samples, there were 28 nucleotide positions identified as variable sites in at least one tissue, for a total of 110 matched tissue variable sites across all samples. There were dam-specific variable sites (e.g. site 434 in dam B; Table 2 and Fig 2), as well as shared variable sites across one or more tissues from two or more dams (e.g. site 3895; Tables 1–3 and Fig 2). This pattern of variation suggests the dam environment, the individual placenta/fetal environments, and likely random chance all contribute to the specific ZIKV variants present within each tissue.

On average, the placentas contained nine variable sites per sample and the fetuses contained seven variable sites per sample. Only one sample, fetus B-2, contained a dominant variant (94.1% at position 4317; Table 2), and this variant was found at 62.6% in the matching placenta. Only 6 of the 110 variable sites across all samples were undetectable in the matched tissue (Tables 1–3), with five of the six undetectable in the fetus, suggesting that these variants either did not transmit from the placentas to the fetuses, or they did not replicate well within the fetus. However, these five sites were all found in dam C, and there was at least one other fetus from the same dam that contained the same variant at >10%, suggesting that lack of transmission of the variant from the placenta in these specific cases is more likely. Only one variable site in one fetus was undetectable in the matched placenta (sample B-1, position 996, Table 2 and Fig 2), suggesting that this variant arose upon replication within that specific fetal environment, or it was present at a very minor frequency in the placenta and was not picked up by sequencing.

These results indicate that while specific frequencies of variants may differ between placentas and fetuses, the overall makeup of variants and ZIKV population structures were very similar between the placenta and fetal virus populations at the early stages of placenta-to-fetus transmission. This suggests that there is little or no selection of specific ZIKV variants during vertical transmission. The similarity in population structure is even more striking considering that the ZIKV populations in the placenta underwent more rounds of replication than those in the fetus (Fig 1A and 1B). Taken together, these results indicate that there is very little restriction of ZIKV transmission between the placenta and fetus and most of the diversity in the ZIKV population is maintained during vertical transmission in the AIR mouse model.

### There is a sharp reduction in ZIKV diversity in most fetal bodies at late stages of ZIKV fetal infection

**Fig 3:**
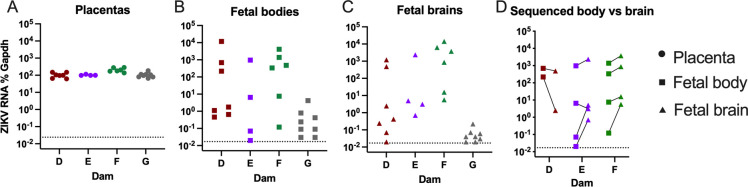
ZIKV RNA levels in placentas, fetal bodies, and fetal heads at 13 dpi during late stages of vertical transmission. Pregnant AIR mice were inoculated at 7 days post-mating. A-C) At 13 dpi, mice were euthanized and tissues analyzed by RT-qPCR for ZIKV RNA in (A) placentas, (B) fetal bodies, and (C) fetal brains. D) ZIKV RNA levels in fetal brains selected for sequencing and the matched body is plotted with lines connecting tissue from the same fetus. Dashed lines denote mock-inoculated levels in the tissues.

Because there appeared to be little restriction of ZIKV variants entering the fetus from the placenta, we next evaluated the ZIKV populations involved in transmission from the fetal periphery to the fetal brain (neuroinvasion). To do this, we inoculated four pregnant AIR mice with ZIKV and compared ZIKV in matched placentas, fetal bodies, and fetal brains at 13 dpi, which was the time point associated with the highest levels of ZIKV RNA in the fetal heads from the previous experiment (Fig 1C). Consistent with the previous results, we observed high levels of ZIKV in all placentas, and a range of viral RNA in the fetal bodies and brains (Fig 3A–3C). To maximize the chances of good sequencing coverage of ZIKV in the fetal brains, we selected brains with high levels of ZIKV RNA from fetuses from dam D, and four fetuses each from dams E and F. The associated fetal bodies had variable levels of ZIKV RNA (Fig 3D).

We performed whole genome sequencing on the ten selected fetal brains and their matched placentas. The fetal bodies were excluded from the whole genome sequencing due to the low ZIKV sequencing coverage of fetal bodies in the previous experiment. Mean ZIKV coverage for the placentas was 75-125x and the brains was 45-126x (S2–S4 Tables). The higher ZIKV coverage in fetal brains compared to fetal bodies, despite similar ct values via qPCR, was likely due to differences in the number of infected vs uninfected cells present in the samples, and thus the proportion of host RNA to ZIKV RNA differed. A higher percentage of reads mapped to ZIKV in the fetal brains (average 1.864%) than in the fetal bodies from the previous experiment (average 0.002%). Variants were identified and reported as described above with sites that differed from the reference in 10–90% of the reads for a given tissue designated “variable sites” and sites with a variant in >90% of reads designated “dominant variants”. We then performed targeted sequencing on these sites and those identified in the first set using the same 13 primer pairs as described above in the matched placentas, fetal bodies, and fetal brains. The targeted sequencing resulted in average coverage of the targeted sites of 173-204x for placentas, 175-223x for fetal bodies, and 164-196x for fetal brains (Tables 4–6).

**Table 4:** Variant frequencies from targeted sequencing for dam D.

							Dam D Targeted					
							D-1			D-2		
	Nucleotide			Amino acid			Plac	Body	BR	Plac	Body	BR
Gene	Pos	Ref	Alt	Pos	Ref	Alt	198x	186x	164x	189x	206x	185x
Capsid	346	T	C	80	I	T	77.7	**100**	**100**	86.2	**100**	**100**
NS1	3104	G	T	999	E	D					11.2	
NS1	3106	A	G	1000	K	R					11.2	
NS2A	3895	C	T	1263	A	V	74.8	**100**	**99.6**	82.0	**100**	**100**
NS2B	4338	A	G	1410	L		10.3					
NS3	5744	A	G	1879	T		13.6					
NS3	6287	T	C	2060	D		10.8		1.1			
NS3	6305	T	G	2066	D	E	14.1		1.1	11.1		
NS5	8603	C	T	2832	A		10.1					
quality score	≥90					variable sites (10–90%)	7	0	0	3	2	0
	*≤89*					**dominant variant (≥90%)**	0	2	2	0	2	2

If matched value of a variable site <1%, left blank

x = average coverage per position of targeted sites

**Table 5:** Variant frequencies from targeted sequencing for Dam E.

							Dam E Targeted											
							E-1			E-2			E-3			E-4		
	Nucleotide			Amino acid			Plac	Body	BR	Plac	Body	BR	Plac	Body	BR	Plac	Body	BR
Gene	Pos	Ref	Alt	Pos	Ref	Alt	196x	190x	177x	191x	194x	178x	196x	183x	174x	187x	175x	189x
Capsid	449	C	T	114	G									9.2	**98.4**		14.7	**97.5**
prM	560	G	A													*8*.*5*	14.3	
NS1	2987	T	A	960	S	R	16.4			18.2			16.9	*6*.*9*		14.8	10.9	
NS2A	3895	C	T	1263	A	V	60.4	**100**	**100**	57	**99.7**	**98.9**	64.9	83.9	1.4	59.3	45.4	1.2
NS2A	4004	A	T	1299	A		13.1		1.1	23.4			17.7	*7*.*6*		13.3	*7*.*5*	1.1
NS2A	4175	C	T	1356	D										13.6			21.6
NS2B	4319	G	A T	1404	M	I	15.8 6.1			13.5 0			17.2 6.0		0 **98.9**	13.3 8.8	10.2 25.4	0 **98.6**
NS3	4823	A	G	1572	G		28.3	**99.1**	**100**	24.5	6.8	30.2	23.5	74.7		17.7	25.8	
NS3	5336	G	A	1743	V									4.4	**98.1**		14.8	**97.2**
NS5	8603	C	T	2832	A		15.7			16.2			26.8			13.8	14.9	
quality score	≥90					variable sites (10–90%)	6	0	0	6	0	1	6	2	1	6	9	1
	*≤89*					**dominant variant (≥90%)**	0	2	2	0	1	1	0	0	3	0	0	3

If matched value of a variable site <1%, left blank

x = average coverage per position of targeted sites

**Table 6:** Variant frequencies from targeted sequencing for Dam F.

							Dam F Targeted											
							F-1			F-2			F-3			F-4		
	Nucleotide			Amino acid			Plac	Body	BR	Plac	Body	BR	Plac	Body	BR	Plac	Body	BR
Gene	Pos	Ref	Alt	Pos	Ref	Alt	204x	199x	177x	203x	223x	196x	175x	196x	178x	173x	183x	169x
Capsid	449	C	T	114	G		70.8	**100**	**100**	69.9	**100**	**100**	73.8	**100**	**100**	74	**99.2**	**100**
NS1	3269	C	T	1054	Y		14.0			17.6						11.4		
NS1	3535	T	C	1143	V	A	18.9			21.3			12.6			18.2	1.9	
NS2A	4175	C	T	1356	D													12.7
NS2B	4317	A	G	1404	M	V	17.1			16.1			13.9			15.7		
NS2B	4319	G	T	1404	M	I	69.1	**100**	**100**	72.9	**100**	**100**	75	**100**	**100**	72.9	**95.8**	**99.6**
NS3	4697	T	C	1530	R		15.4			11.3								
NS3	5336	G	A	1743	V		75.0	**100**	**100**	80.8	**100**	**100**	79.4	**100**	**100**	67.5	**98.2**	**98.1**
NS3	5460	A	G	1785	I	V			23.9									
quality score	≥90					variable sites (10–90%)	8	0	0	7	0	0	5	0	0	6	0	1
	*≤89*					**dominant variant (≥90%)**	0	3	3	0	3	3	0	3	3	0	3	3

If matched value of a variable site <1%, left blank

x = average coverage per position of targeted sites

**Fig 4:**
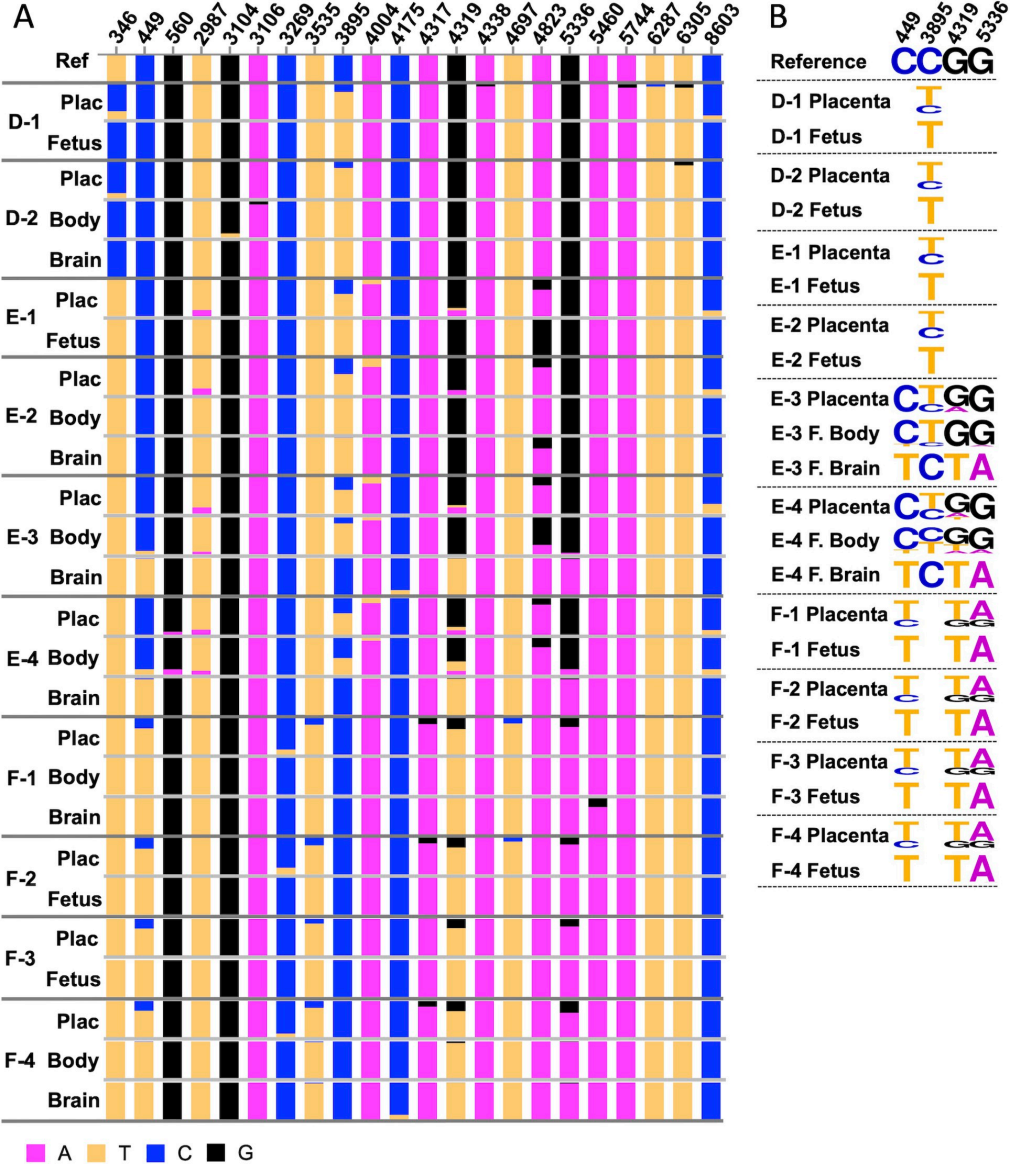
Graphical representation of variable sites and dominant variants from targeted sequencing of late stages of vertical transmission and neuroinvasion. A) Sequencing data summarized in Tables 1–3 is shown. All variable sites across all three dams are plotted. Ref indicates the reference nucleotide, and proportions of reference vs. alternate allele are represented in each bar graph. Plac = placenta, Fetus = representation of both fetal body and fetal brain variants as they were identical. Matched tissues are separated by light gray lines, different samples are separated by dark gray lines. B) Sequence logos for the dominant variants found across multiple fetuses in multiple dams for the four sites contained in the two dominant variant haplotypes are shown. The site position is listed at the top, and the frequency of the nucleotide is represented by the size of the base letter. Reference refers to the inoculum reference nucleotide. For the eight matched samples where the shared dominant sites were the same in both the fetal body and fetal brain, these ZIKV populations are summarized as a single “Fetus” logo. For the two samples that did not have matching fetal body and fetal brain populations, the ZIKV genomes are split into separate Fetal body (F. Body) and Fetal Brain (F. Brain) logos. The nucleotide frequency was not depicted if it was not part of the sample’s haplotype and was 100% reference.

Variant frequencies comparing the whole genome and targeted approaches for the placentas and fetal brains are reported for each dam in S2–S4 Tables. Overall, the sequencing results matched between the whole genome and targeted approaches for both the placentas and the fetal brains. Most variants that reached the ≥10% threshold in the whole genome approach also reached this threshold in the targeted. There were eight instances across the twenty samples where the ≥10% threshold was reached in one method and not the other, but the variant was still detectable in the other method (S2–S4 Tables). There were only five instances where a variant was undetectable in one method (S2–S4 Tables), but in four of the five instances the quality score for the undetected variant was poor, and therefore likely missed due to poor sequencing quality. Overall, these results confirmed both methods identified variants at similar frequencies and confirmed the variant calls. Variant frequencies from the targeted sequencing comparing placentas, fetal bodies, and fetal brains from each dam are shown in Tables 4–6 and graphically displayed in Fig 4A.

As with the first set of sequencing, the placentas represented a moderate heterogenous population, with an average of six variable sites per placenta, compared with nine in the first set (Tables 1–6). This may represent a modest decrease in diversity within the placenta at this later stage of infection. But as with the first set, there were no dominant variants present in any of the placentas (Tables 4–6). However, unlike the very similar population structures observed in the placentas and fetal bodies during early stages of ZIKV infection in the fetuses, (Tables 1–3), the ZIKV populations in the fetal bodies at this later stage of fetal infection were largely homogenous (Tables 4–6 and Fig 4A). For eight of the ten fetal bodies, the ZIKV populations in the placentas had more variable sites than the fetal bodies, whereas the fetal bodies had more dominant variants (Tables 4–6).

The two fetal body samples from dam D both contained two dominant variants at positions 346 and 3895, and only two variable sites in D-2 (Table 4). Both the 346 and 3895 dominant variants encoded nonsynonymous changes, I80T in Capsid and A1263V in NS2A, respectively. The four fetal body samples from dam F all contained three dominant variants at positions 449, 4319, and 5336, and no variable sites (Table 5). Only one of the three dominant variants, position 4319, encoded a nonsynonymous change of M1404I in NS2B, the 449 and 5336 sites both encoded synonymous mutations in the Capsid and NS3 proteins, respectively. Of the four fetal body samples from dam E, two (E-1 and E-2) contained a dominant variant at position 3895, with sample E-1 containing an additional dominant variant at site 4823, encoding a synonymous mutation in NS3 (Table 5).

There were two fetuses, both from dam E, where the fetal body ZIKV populations did not contain dominant variants. In sample E-3, the fetal body ZIKV population did have a reduction in diversity compared to the placenta (two variable sites versus six, respectively), but it comprised two variable sites at positions 3895 and 4823 and no dominant variants (Table 6). Interestingly, the two variable sites in the fetal body at positions 3895 and 4823 were found at high frequencies (83.9% and 74.7%, respectively), and were dominant variants in other fetal bodies from the same dam (Table 6). Therefore, it is possible that these variants were trending towards becoming the dominant variants in this fetal body. The second fetal body that did not have a homogenous population was sample E-4. The ZIKV population in this fetal body was heterogenous, with nine variable sites and no dominant variants (Table 6). This particular fetal body population was more like the matched placenta ZIKV population and similar to what was observed in the earlier stages of transmission from placenta to fetus (Tables 1–3), suggesting that perhaps this sample represents an intermediate stage of vertical transmission.

Overall, there was a small reduction in ZIKV diversity in the placentas and a sharp reduction of ZIKV diversity in most fetal bodies at this later stage of ZIKV infection. This observation suggests that after viral entry into the fetus from the placenta, selection pressures within the fetus result in the homogenous population.

### ZIKV populations in fetal brains are homogenous and similar to the fetal bodies

We next analyzed the ZIKV populations in the fetal brains and compared these with the matched fetal bodies. Like the fetal bodies at the late stage of infection, the ZIKV populations in the fetal brains were largely comprised of homogenous populations. All 10 fetal brains contained dominant variants, compared to 8 of 10 fetal bodies and 0 of 10 placentas (Tables 4–6). Five of the fetal brains contained a single variable site (E-2, E-3, E-4, F-1, and F-4). Four of the five variable sites were undetectable in the matched fetal body, suggesting these variants arose spontaneously within the fetal brains (Tables 5 and 6). The other five fetal brains did not contain any variable sites (D-1, D-2, E-1, F-2, and F-3). Of the eight fetal bodies that contained mostly homogenous populations with dominant variants, the matched fetal brains shared the same dominant variants (Tables 4–6).

Of the two fetal body samples that did not contain dominant variants, E-3 and E-4, their matched fetal brains contained three dominant variants at positions 449, 4319, and 5336 (Table 5). Interestingly, not only did these two fetal brains contain dominant sites that their matched fetal bodies did not, the fetal body populations did not closely resemble the fetal brain population. For fetus E-3, the variants present at the highest frequency in the body, 3895 (83.9%) and 4823 (74.4%), were only found in the matched fetal brain at 1.4% and 0, respectively. The dominant sites in the E-3 fetal brain at positions 449, 4319, and 5336 were only detected in the matched fetal body at frequencies of 9.2, 0, and 4.4%, respectively. The E-4 fetal body contained the matched brain’s three dominant variants at frequencies of 14.7, 25.4, and 14.8%, however the fetal body population also contained six other variable sites with variant frequencies of 10.2–45.4%. The dissimilarity in these two fetuses between their body and brain ZIKV populations is surprising considering the other eight fetal bodies and brains have identical dominant variants.

### Evidence of possible selection of certain ZIKV variants

The homogenous ZIKV populations in the fetal bodies and brains may arise due to genetic drift, purifying selection, or positive selection pressures on variants with beneficial transmission or replication phenotypes. Traditional tests of selection, such as the ratio of nonsynonymous to synonymous substitutions (dN/dS ratios), are not appropriate in closely related populations and ignore the non-silent nature of some synonymous changes on RNA secondary structure, which can alter replication and translation of viruses and affect their fitness in different environments [23]. An alternative method for estimating selection of viral variants relies on estimating changes in variant frequency at multiple sequential time points [24]. Because we could not take sequential samples from the same tissues from the same mouse over time, our samples did not fit the criteria of sequential sampling. Other methods of estimating viral evolution within populations require the reconstruction of viral haplotypes, which is generally poor from RNASeq data, especially when there is a low level of variation [25].

Therefore, we leveraged the fact that we had sequence data from multiple fetuses from multiple dams to identify common ZIKV variants in the fetuses across multiple dams. While variants unique to a single fetus or a single dam were likely acquired due to random chance or dam-specific selection pressures, the presence of specific dominant variants in the fetuses across multiple dams could be suggestive of selection for those variants. From the 10 fetal brains and bodies from the three dams (D, E, and F), there were four sites containing dominant variants within fetal bodies and brains from more than one dam. These four shared dominant variants segregated into two overall “haplotypes” (we could not accurately reconstruct haplotypes from the short sequencing reads, but based on the homogenous populations containing certain variants, we can infer they occur together in the genome, and we are therefore using the terminology “haplotype” here). The first haplotype contained a C3895T mutation (“single variant haplotype”), which results in a nonsynonymous mutation of A to V at polyprotein amino acid 1263 in the NS2A protein. The second haplotype contained three variants comprised of C449T, G4319T, and G5336A (“triple variant haplotype”). The mutations at C449T and G5336A are synonymous, whereas the G4319T results in a nonsynonymous mutation of M to I at polyprotein amino acid 1404 in the NS2B protein. The ZIKV populations in the two matched fetal brains and bodies from dam D were all comprised of the same single variant haplotype (Table 4). The ZIKV populations for all four matched fetal brains and fetal bodies from dam F were comprised of the triple variant haplotype (Table 6). Interestingly, of the four fetuses from dam E, two matched brain and bodies contained the single variant haplotype (samples E-1 and E-2; Table 5). The other two fetal brains from samples E-3 and E-4 contained the triple variant haplotype. However, the E-3 and E-4 fetal bodies did not contain the triple variant haplotype, at least not as a majority proportion of the population (Tables 5 and 6). Interestingly, the E-3 fetal body contained C3895T of the single variant haplotype in 84% of the reads, suggesting the ZIKV population in this fetus may have been trending towards a homogenous population comprised of the single variant haplotype. The E-4 fetal body also contained the C3895T variant of the single variant haplotype at a frequency of ~45%, however it also contained the variants that comprise the triple variant haplotype at frequencies of 14.7–25.4% along with six other variants at frequencies of 7.5–25.8%.

The presence of the same haplotypes dominating the population in multiple fetuses and fetal brains from multiple dams suggests these haplotypes may be the result of positive selection rather than purifying selection or genetic drift. However, if the variants comprising the haplotypes were present in the starting population at high frequencies, it likely would be the result of random chance. From the first set of samples comparing the ZIKV populations during early stages of vertical transmission in the placenta and fetal bodies, we established that the fetal body populations contained similar variants at similar frequencies between the placenta and fetal bodies. We therefore used the variant frequencies in the matched placenta populations as a proxy for the starting population within the fetus at the early stage of vertical transmission. The comparison of the four dominant variants found across multiple dams in the placentas and fetal brains/bodies is visually summarized in Fig 4B.

Of the four fetal brains/bodies containing the 3895 single variant haplotype (D-1, D-2, E-1, E-2), this haplotype was present in the majority of sequences from all four of their matched placentas (variant frequency 57–82%; Fig 4B and Tables 4 and 5). Therefore, this haplotype likely was present at a high frequency in the fetus at the time of transmission to the brain, and may have become the dominant variant in the brain simply due to chance. The 449, 4319, and 5336 triple variant haplotype found in six fetal brains (E-3, E-4, F-1, F-2, F-3, and F-4) was also present in the majority of sequences from the four placentas from dam F (variant frequency 68–81%; Fig 4B and Tables 5 and 6). However, in the E-3 and E-4 placentas, the 449 and 5336 variants were not detected in any of the sequences, and the 4319 variant was only present in 6–8.8% of the sequences (Table 5 and Fig 4B). In the matched fetal bodies for these samples, the variants were detected at higher frequencies than in the placentas but were not detected in the majority of ZIKV sequences in the fetal bodies (frequencies of 4.4–25.4%; Table 5 and Fig 4B). The increase in frequency from the placenta to the fetal body to the fetal brain suggests that these variants may have been selected for in the fetal tissues, however we cannot definitively make this conclusion.

## Discussion

The results of our study indicate that in the AIR mouse model there is little to no restriction of ZIKV variants initially entering the fetus from the placenta during vertical transmission, but a strict reduction of ZIKV diversity in both the fetal bodies and fetal brains at later stages of fetal infection and neuroinvasion. The observation that the fetal tissues were dominated by two primary viral variant haplotypes across multiple fetuses from separate dams suggests possible selection for specific variants in the fetal environment. We cannot make definitive claims about selection based on variant frequencies in tissues taken on the same day that have undergone different lengths of replication, and we also cannot rule out the possibility that these variants arose due to chance. However, the observation that the triple variant was detected in fetuses from multiple dams, was either undetectable or present at a minor frequency in the matched placentas of two different fetuses, increased in the fetal bodies, and then became the dominant variant in their brains is suggestive of selection.

Interestingly, the mutation at 4319 in the triple variant results in a M1404I mutation previously identified and characterized in a study of pregnant rhesus macaques [13], but it was determined not to be associated with fetal death. The authors then examined the mutation in pregnant CD-1 mice using a ZIKV infectious clone. They found that the 1404I mutation was associated with slightly higher replication in maternal tissues at later stages of infection and higher levels of vertical transmission [13]. Interestingly, reversion to 1404M occurred in most fetuses, and very little neuroinvasion occurred. This contrasts with our results, which observed the 1404I mutation in five of eleven fetuses at frequencies of ~38–85% in the early stages of vertical transmission (Tables 1 and 3) and 11 of 20 fetal bodies and brains in the late stages of vertical transmission (Tables 4–6). We also observed an M1404V amino acid change from the A4317G nucleotide mutation in five of eleven fetuses at frequencies of ~33–94% at the early stage of vertical transmission, but not in fetuses at the late stages of vertical transmission (Tables 2–3). The differences in the presence of the M1404I mutation in the previous study and ours may be due to the different mouse models, as the CD-1 mouse model had low levels of vertical transmission to the fetus and almost no neuroinvasion [13], whereas the AIR mouse model has high levels of vertical transmission and neuroinvasion.

A second amino acid change at position 1143 was previously identified in the brain of a human fetus aborted at 29 weeks due to ZIKV congenital disease [26]. The authors identified a M1143V polymorphism, which differed from the French Polynesia/Brazil reference strain. Our virus stock contained V1143 as the reference amino acid, and we observed a nonsynonymous V1143A mutation in eight of the eleven placenta/fetus pairs at early stages of vertical transmission and in four placentas and one fetal body at the late stages of vertical transmission (Tables 1–3 and 6). The fact that we identified mutations at amino acids previously reported in macaque, mouse, and human ZIKV infections suggests that despite the immunocompromised nature of the AIR mouse model, there is some degree of similarity in selective pressures across ZIKV in multiple infection contexts.

At the late stages of vertical transmission, there were two fetal bodies that did not match their brain ZIKV population. Interestingly, the two fetuses with less homogenous ZIKV populations were both from dam E, which were one embryonic day younger than the fetuses from the other two dams in this set (~E17.5 for dam E vs. ~E18.5 for dams D and F). They also had the two lowest levels of ZIKV RNA by qPCR of any of the sequenced fetal bodies (Fig 3D). It is of note that the matched fetal brains for these two fetuses had higher viral RNA levels compared to the fetal bodies (Fig 3D), which is in contrast to what we observed in the previous experiments (Fig 1B and 1C). It is possible that the ZIKV populations from this dam, or these two particular fetuses, had less replication time within the fetal bodies than the other fetuses in this set and may represent a more intermediate stage of infection. If we had sequenced these tissues at a later day, the fetal body ZIKV populations from E-3 and E-4 may have become a more homogenous population like observed in the other fetal bodies. However, since we cannot take sequential samples from the same fetal tissues over time, we cannot confirm this.

The observed reduction in ZIKV diversity in both the fetal bodies and fetal brains at the late stages of ZIKV infection could arise by the reduction in ZIKV diversity first occurring in the fetal body prior to neuroinvasion, or by the selection of certain ZIKV variants during entry into the brain. Subsequent selection pressures may then reduce the diversity in the fetal body after neuroinvasion. That eight of the ten matched fetal bodies and brains had identical dominant variants implies that the reduction in ZIKV diversity first occurred in the fetal periphery prior to neuroinvasion to the brain. However, the observation that two fetal bodies and matched fetal brains had dissimilar ZIKV populations suggests that only a small founder population entered the brain prior to or during the reduction of ZIKV diversity in the fetal periphery. It is also possible that the ZIKV populations in the brains contribute to the homogenous populations in the fetal bodies by dissemination. While mice develop a functional and selective blood brain barrier by E15 [27], mice do not develop a meningeal lymphatic system for drainage out of the brain until after birth [28]. Viruses, including ZIKV, can utilize the meningeal lymphatic system in adult mice to spread to the periphery from the CNS [29]. It is possible that without a fully developed meningeal lymphatic system, drainage of molecules from the brain is less restricted in the fetal mouse. Therefore, the decrease in diversity in ZIKV populations in the fetal bodies may be due to the dominant fetal brain variants disseminating out of the brain and replacing the population in the periphery. This brain-seeding mechanism could also potentially explain the difference in ZIKV populations in the fetal bodies that do not match their fetal brains. If those samples were from an intermediate stage of infection, the virus from the brain would not have had the same amount of time to escape the brain and contribute to the peripheral population.

The immunocompromised nature of our mouse model may influence the dynamics of vertical transmission. ZIKV effectively antagonizes the human type I IFN response by targeting human signal transducer of transcription 2 (STAT2) to inhibit a productive type I IFN response [30]. However, ZIKV does not antagonize mouse type I IFN which renders wild type mice largely unsusceptible to ZIKV infection and disease [31]. To overcome this barrier to disease, a variety of mice with deficiencies in the type I IFN response have been used for ZIKV infection studies. We used the previously established anti-IFNAR1 treated *Rag1-/-* (AIR) mouse model of ZIKV vertical transmission [18]. These mice are treated with an anti-IFNAR1 antibody to suppress but not ablate the type I IFN response to mimic human infection. Although these mice also lack B and T cells, we selected this model because it was associated with higher fetal viability than the *Ifnar-/-* x WT model [16,17]. The adaptive immune response has been shown to be important for preventing the dissemination of ZIKV to the brains and testes of non-pregnant adult mice [18], and it is therefore possible that the lack of B and T cells in the dam could influence the viral variants present in the placenta and their ability to vertically transmit to the fetus. However, as adaptive immune responses do not develop until after birth in the mouse, there would be no effect of lymphocyte deficiency on selection in the fetus. Our observations of two amino acid changes previously identified in rhesus macaques and a human fetus suggests there may be conserved selective pressures to common regions of the ZIKV genome across multiple species, regardless of immune status.

We observed nearly 100% fetal infection in the AIR model, whereas in humans only ~5% of ZIKV pregnancies result in fetal harm [32,33]. There is likely a stricter bottleneck both into the fetus and into the fetal brain due to additional immune selection pressures in immunocompetent humans than in the AIR mouse model. Even so, ZIKV may follow a similar pattern of transmission in humans, with a loose/moderate bottleneck into the fetus and a very strict bottleneck into the fetal CNS. This may, in part, explain the ~5% incidence of CZS in humans. ZIKV may vertically transmit to fetuses in more than 5% of cases, and indeed this has recently been supported in an analysis of mean risk of vertical transmission in humans [34]. Therefore, the development of ZIKV-related microcephaly and birth defects in humans may be due to the ability of certain ZIKV variants, or the interactions between multiple variants, to cross two bottlenecks: from the placenta to the fetal periphery, then to the fetal brain.

## Methods

### Ethics statement

All animal work was conducted in compliance with the guidelines of and under a protocol approved by the Rocky Mountain Labs (RML) Institutional Animal Care and Use Committee (IACUC, Protocol #2018-001E). All applicable international, national, and/or institutional guidelines for the care and use of animals were followed.

### Cells and virus

The human microcephaly isolate ZIKV Paraiba strain was provided by Steve Whitehead (NIAID). Virus stocks were passaged 2 times in C6/36 cells (ATCC) maintained in minimum essential medium supplemented with 10% fetal bovine serum, 2mM glutamine, 1x nonessential amino acids and 1% penicillin/streptomycin. Virus titers of supernatants were determined by plaque assay using Vero cells as previously described [18]. All cell culture reagents were obtained from Gibco (https://www.biosciences.ie/gibco).

### Treatment, infection and tissue collection from ZIKV infected mice

*Rag1*^-/-^ mice (Jackson Laboratories), which are deficient in B and T lymphocytes, were maintained on a C57BL/6 background in a breeding colony at RML. For these experiments, pregnant anti-IFNAR1 treated *Rag1*^*-/-*^ (AIR) mice were generated as previously described [16] with a few modifications. At 8–12 weeks old, *Rag1*^-/-^ female mice were time-mated with age-matched *Rag1*^*-/-*^ males for 3 days. Six days after the initial pairing (embryonic day (E) 3–6), pregnant AIR mice were treated intraperitoneally (i.p.) with 1mg of anti-IFNAR1 clone MAR1-5A3. The following day (E4-7), mice were inoculated i.p. with 10^4^ plaque forming units (PFU) of ZIKV in a volume of 200 μl/mouse diluted in PBS. Three days post infection (3 dpi, E7-10), mice were again treated i.p. with 1mg of MAR1-5A3. Some pregnant mice that were euthanized at 7dpi (E11-14) and had tissues collected received no further treatments. Others that were aged out as late as 14dpi (E17-20) received an additional 2 treatments of 0.5mg of MAR1-5A3 on 7dpi (E11-14) and 11dpi (E15-E18).

At the indicated time points, pregnant mice were humanely euthanized according to the IACUC protocol and a midline incision was made through the abdominal wall along the length of the peritoneal cavity to expose the gravid uterus. Then, as quickly as possible, the uterus was externalized, and the lower aspect of the cervix was separated from the vagina. Subsequently, the mesometrium was cut at the head of each uterine horn near the ovary to remove the entire uterus from the peritoneal cavity. In all cases, fetuses with their associated placenta were individually dissected from the uterus, and matched placenta and fetal tissues were collected in separate tubes and snap frozen in liquid nitrogen for further processing and analysis. For experiments involving timepoint analysis of ZIKV transmission, fetuses at ~E11 were collected whole and intact. Of 11 fetuses at ~E14, 8 were collected whole and three were decapitated to separate the fetal body from the head. All fetuses older than ~E14 were decapitated to separate the fetal body from the head. For experiments comparing placenta and fetal brain ZIKV populations, matched placentas and fetal brains were collected.

### Isolation of RNA from placenta and fetal tissues

RNA was isolated from snap-frozen tissues by homogenization in TRIzol on a bead mill at 5300 rpm 2x for 25 seconds, with a 5 second pause in between. Placentas, fetal heads, and fetal brains were homogenized in 1 ml TRIzol, and whole fetus and fetal bodies were separated into two tubes and homogenized with 1 ml of TRIzol in each tube. Chloroform extractions were performed by adding 200ul chloroform to each tube, and centrifuging at 12,000xg for 15 minutes at 4°C. Isopropanol precipitations were performed on the aqueous phase; 600ul isopropanol was added to each sample, and samples were incubated at room temperature for 1 hour, then pelleted by centrifugation at 12,000xg for 10 minutes. The supernatant was removed and pellets were washed with 1ml 70% EtOH at 7,600xg for 5 minutes. The supernatant was removed and pellets were air-dried for 5 minutes then resuspended in 50ul nuclease-free water, and duplicate fetal body samples were combined. Aliquots (5ul) were removed for use in qPCR, and the remainder was frozen at -80°C for use in RNASeq.

### RT-qPCR of placenta and fetal tissues

The 5ul aliquots taken from the TRIzol extractions were treated with DNase I (Ambion) per manufacturer’s instructions, then immediately cleaned up with the Zymo cleanup kit per manufacturer’s instructions, and were eluted in 50ul nuclease-free water. cDNA was synthesized using the iScript cDNA synthesis kit (BioRad) according to the manufacturer’s instructions. RT-qPCR was performed on samples as previously described [35] for *Gapdh* and ZIKV RNA. The primers used were Gapdh.2-152Forward 5’-TGCACCACCAACTGCTTAGC-3’ and Gapdh.2-342 Reverse 5’-TGGATGCAGGGATGATGTTC-3’, and ZIKV.8008Forward 5’-AAGCTGAGATGGTTGGTGGA-3’ and ZIKV.8121Reverse 5’-TTGAACTTTGCGGATGGTGG-3’ [16]. ZIKV RNA was calculated as the percent difference in threshold cycle (Δ*C*_*T*_ = (Gapdh C_T_)–(ZIKV C_T_)). ZIKV RNA was plotted as the percentage of *Gapdh* mRNA.

### Whole genome sequencing of ZIKV variants via RNASeq

For analysis of ZIKV populations in placentas and fetal bodies, 12 matched tissue, 4 each from 3 separate dams (A, B, and C) were selected for RNASeq. All placentas and fetal bodies were determined to have high levels of ZIKV RNA by qPCR, but associated heads had low ZIKV RNA. For analysis of ZIKV populations in placentas and fetal brains, 10 matching placentas and brains from fetuses that were viable at the time of necropsy and with high levels of ZIKV RNA in their brains were selected for RNASeq. Two fetal brains from dam D, and 4 fetal brains each from dams E and F were used. Samples were purified using Qiagen’s Viral RNA extraction kit, with some modifications. 560 ul Buffer AVL (without carrier RNA) was added to the remaining TRIzol-extracted RNA not used in RT-qPCR analysis and mixed by 15 seconds of pulse-vortex and incubated at RT for 10 minutes. Samples were then added to QiaShred columns and spun at 20,000xg for 2 minutes, then 560ul 100% EtOH was added to the sample and pulse-vortexed for 15 seconds. The solution was added to the QIAamp Mini column in two additions, each centrifuged at 6000xg for 1 minute. Then, 250ul Buffer AW1 was added to each column and centrifuged at 6000xg for 1 minute. An on-column DNase digestion was performed by adding 10ul DNase I plus 70ul of the provided Buffer RDD (Qiagen) to each column and incubating 15 minutes. An additional 250ul Buffer AW1 was added to the columns and centrifuged at 6000xg for 1 minute, then 500ul Buffer AW2 was added and centrifuged at 20,000xg for 3 minutes. The columns were then placed in new collection tubes and spun for an additional minute at 20,000xg. Samples were eluted in 60ul Buffer AVE at 6000xg 1 minute. At this point, RNA from placentas, fetal heads, and fetal brains were stored at -80°C. Due to residual DNA in fetal bodies, the eluted RNA from fetal bodies was treated with additional DNase by adding 2.5ul DNase I and 10ul RDD Buffer to the eluted fetal body RNA, then bringing the volume to 100ul with nuclease-free water. Samples were incubated at room temp for 10 minutes, then 560ul 100% EtOH added. Samples were added to QIAamp Mini columns as described above, washed with 500ul Buffer AW1 at 6,000xg 1 minute, and washed with 500ul Buffer AW2 and eluted in 60ul AVE Buffer as described above. Eluted RNA was stored at -80°C.

For RNASeq runs comparing placentas and fetal bodies, 2ug of each sample, excluding the Zika virus inoculum, were host ribosomal RNA-depleted using the Ribo-Zero H/M/R Gold kit (Illumina, San Diego, CA). The depleted RNA was purified with Agencourt RNAClean beads (Beckman Coulter, Indianapolis, IN) and eluted in 50 ul of water. The attempt was made to further deplete the samples of host mRNA by following the oligo-dT bead binding steps in the TruSeq Stranded mRNA Seq kit deviating from the protocol by saving the supernatant after the bead binding step. The supernatant and ZIKV inoculum sample were purified with Agencourt RNAClean beads and eluted in the TruSeq Fragment, Prime, Finish mix. Libraries were prepared following the TruSeq Stranded mRNA Sample Prep protocol starting at the Elution 2 –Frag- Prime step without modification. The final libraries were assessed on a 2100 BioAnalyzer using the DNA1000 chip (Agilent, Santa Clara, CA) and quantified using the Kapa Quantification Kit (Kapa Biosystems, Wilmington, MA) for Illumina Sequencing. Libraries were diluted to 2 nM, pooled equally and run on the NextSeq sequencing instrument as 2 X 150 bp reads.

For RNASeq runs comparing placentas with fetal brains, 2 ug of each sample were host ribosomal RNA depleted using the Ribo-Zero rRNA Removal H/M/R Gold kit (Illumina, San Diego CA). The depleted RNA was purified with Agencourt RNAClean beads (Beckman Coulter, Indianapolis, IN) and eluted in Illumina’s Fragment, Prime, Finish mix from the TruSeq Stranded mRNA Sample Prep kit. Libraries were prepared following the TruSeq Stranded mRNA Sample Prep protocol starting at the Elution 2 –Frag- Prime step without modification. The final libraries were assessed on BioAnalyzer DNA1000 chips (Agilent Technologies, Santa Clara, CA) and quantified using the Universal Kapa Quantification Kit for Illumina Platforms (Roche, Basel Switzerland). Based on data from an Illumina NextSeq High Output 75 cycle run, the samples were pooled to target 100X coverage of the viral genome and sequenced using a NextSeq High Output 300 cycle kit generating roughly 377 M reads.

### Targeted sequencing of ZIKV variants via RNASeq

Targeted sequencing was performed by designing primers to cover 31 sites with variant nucleotides identified at ≥10% frequency from whole genome sequencing. Thirteen primer pairs with amplicon lengths of 400–411 bp were designed and named for the start and end positions of the amplicon in the genome and are summarized in S5 Table.

Select placental, fetal brain, and fetal body samples were amplified with the thirteen primer pairs specific for ZIKV, separated into two multiplexed pools of odd and even primer sets. Following the ARTIC protocol for SARS-CoV-2 sequencing (https://www.protocols.io/view/ncov-2019-sequencing-protocol-bbmuik6w?version_warning=no), eleven microliters of RNA diluted 1:10 was used to generate first strand cDNA. After first strand synthesis, 2.5 uL of cDNA was used for PCR amplification for either 27 or 33 cycles depending on Zika Ct values with both the Odd and Even primer pool sets. The Odd/Even PCR products were assessed on BioAnalyzer 2100 DNA1000 chips (Agilent Technologies, Santa Clara, CA) for concentration and sizing. Sequencing ready libraries were generated using 800 ng (or all the PCR product if it did not reach that threshold) of pooled Odd/Even multiplexed products for each sample using the TruSeq DNA PCR-Free Library Preparation Kit, beginning at End-Repair (Illumina, San Diego, CA). Size selection was omitted after end-repair and one cleanup using 1.8X AMPure beads was performed. Final libraries were assessed on BioAnalyzer DNA1000 chips and quantified using the Kapa SYBR FAST Universal qPCR kit for Illumina sequencing (Kapa Biosystems, Boston, MA) on the CFX384 Real-Time PCR Detection System (Bio-Rad Laboratories, Inc, Hercules, CA). Paired-end 2 x 250 bp cycle sequencing was completed on the Illumina MiSeq using Nano 500 cycle chemistry.

### Sequence data analysis

Raw, paired-end sequence FASTQ files were trimmed of adapter sequences using cutadapt version 1.12 with Python 2.7 and quality trimmed and filtered using fastq_quality_trimmer and fastq_quality_filter tools from the FASTX-Toolkit, v 0.0.14 (http://hannonlab.cshl.edu/fastx_toolkit/). Singletons were removed and trimmed reads were sorted in coordinate order using a custom Perl script.

Quality-trimmed and sorted reads were aligned to the ZIKV *Paraiba* reference using Bowtie 2, version 2.2.9, with options -X 1000,—no-mixed and—no-unal [36]. Aligned Sequence Alignment/MAP (SAM) files were converted to Binary Sequence Alignment/Map (BAM) format and sorted and indexed using SAMTOOLS version 1.8 [37]. Read duplicates were removed from the BAM files using MarkDuplicates in Picard version 2.18.7 (http://broadinstitute.github.io/picard/).

Variants were detected across all samples using GATK, v4.0, HaplotypeCaller with–emitRefConfidence option to generate genomic variant call format (gVCF) files [21]. Next, joint genotyping was performed and the raw data were formatted as variant call format (VCF) using GATK’s GenotypeGVCFs. Sample sequencing metrics were computed using Picard’s CollectWgsMetrics tool. Variants identified using GATK were manually confirmed in IGV, and those with an AVF ≥10% were reported. GATK is the standard tool for variant detection in NCBI’s SARS-CoV-2 variant calling pipeline [38].

In the placenta to fetal body comparisons (dams A, B, and C), because the fetal bodies had low sequence coverage (5-14x), to accurately compare ZIKV populations, reads from the four fetal bodies and placentas from each dam were combined, bringing the fetal body coverage to 33-46x, and placenta coverage to >3000x. Sequences were aligned to Paraiba reference strain and variants were detected as described above, and variants were manually confirmed in IGV [22]. Predicted amino acid substitutions were evaluated in MacVector. Fig 3 sequence logo schematics generated by Berkley’s WebLogo tool [39].

## Supporting information

S1 Table Variant frequencies in inoculum and whole genome sequencing for combined placentas and fetuses from set 1 evaluating ZIKV diversity during early stages of vertical transmission. (XLSX)

S2 Table Variant frequencies in whole genome vs. targeted sequencing evaluating ZIKV diversity in late stages of vertical transmission for matched placentas and fetal brains from Dam D. (XLSX)

S3 Table Variant frequencies in whole genome vs. targeted sequencing evaluating ZIKV diversity in late stages of vertical transmission for matched placentas and fetal brains from Dam E. (XLSX)

S4 Table Variant frequencies in whole genome vs. targeted sequencing evaluating ZIKV diversity in late stages of vertical transmission for matched placentas and fetal brains from Dam F. (XLSX)

S5 Table Primers used for targeted sequencing and their corresponding start and stop positions in the ZIKV genome. (XLSX)
